# Editorial Comment: Gubernaculum Testis and Cremasteric Vessel Preservation during Laparoscopic Orchiopexy for Intra-Abdominal Testes: Effect on Testicular Atrophy Rates

**DOI:** 10.1590/S1677-5538.IBJU.2021.05.06

**Published:** 2021-07-23

**Authors:** Luciano A. Favorito

**Affiliations:** 1 Universidade do Estado do Rio de Janeiro - Uerj Unidade de Pesquisa Urogenital Rio de JaneiroRJ Brasil Unidade de Pesquisa Urogenital - Universidade do Estado do Rio de Janeiro - Uerj, Rio de Janeiro, RJ, Brasil

Luis H Braga 1, 2, Forough Farrokhyar 3, Melissa McGrath 1, 2, Armando J Lorenzo 4, 5

J Urol. 2019 Feb;201(2):378-385.

## COMMENT

In this important paper the authors shows that the Gubernaculum sparing laparoscopic orchiopexy is a feasible alternative to conventional laparoscopic Fowler-Stephens orchiopexy. This study shows this technique preserves an additional vascular supply to the testis (cremasteric, vessels and deferential arteries) during the laparoscopic orchiopexy. During the abdominal stage of testicular migration the testes migrate from the abdomen to the internal inguinal ring. The gubernaculum has an important role in this process. During the inguinoscrotal stage the gubernaculum approaches the inguinal region distally and after the testes crosses the external inguinal ring the gubernaculum migrates across the pubic region to reach the scrotum (1,2). In an experimental study with 32 human fetuses (3) was demonstrated that the fetal testicle is always irrigated by at least 3 arteries (testicular, cremasteric and deferential) in almost 80% of cases and in the other 20% of the cases there were only 2 arteries irrigating the abdominal testis and the authors shows in this paper that preservation of additional vascular supply to the testis (cremasteric vessels and deferential artery) may translate into improved testicular survival rates following laparoscopic orchiopexy.
